# Fouling‐Free Inertial Microfluidic Cell Clarification for Lentivirus Manufacturing

**DOI:** 10.1002/bit.70262

**Published:** 2026-06-07

**Authors:** Dohyun Park, Alexander Bevacqua, Mingyang Cui, Marina Nichols, Jianzhu Chen, Jongyoon Han

**Affiliations:** ^1^ Research Laboratory of Electronics Massachusetts Institute of Technology Cambridge Massachusetts USA; ^2^ Department of Biological Engineering Massachusetts Institute of Technology Cambridge Massachusetts USA; ^3^ Koch Institute for Integrative Cancer Research Massachusetts Institute of Technology Cambridge Massachusetts USA; ^4^ Department of Physics Boston College Chestnut Hill Massachusetts; ^5^ Department of Biology Massachusetts Institute of Technology Cambridge Massachusetts USA; ^6^ Department of Electrical Engineering and Computer Science Massachusetts Institute of Technology Cambridge Massachusetts USA

## Abstract

Lentiviral vectors (LVVs) are widely used as gene‐delivery vehicles in cell and gene therapy, enabling stable integration of transgenes into target cells. However, efficient clarification of LVV harvest remains a major bottleneck in large‐scale manufacturing, as conventional depth filtration often results in significant product loss. Here, we introduce a fouling‐free microfluidic cell clarification (MCC) system that enables high‐efficiency LVV recovery. By precisely controlling the harvest flow rate and adjusting the recirculated cell density by adding buffer, we established a quantitative relationship between clarification efficiency and throughput. Under optimized conditions, the MCC achieved > 95% product recovery while maintaining > 95% cell removal efficiency. The MCC process closely followed the theoretical recovery model assuming negligible product loss within the device. Across three independent batch operations, apparent average LVV recovery remained substantially higher than that of a benchmark process employing a cellulose depth filter‐based process. Scalability tests demonstrated consistent performance at flow rates up to 1.5 L/h without loss of clarification efficiency, demonstrating its potential for large‐scale biomanufacturing. Beyond LVVs, the MCC platform is broadly applicable to cell‐based modalities that secrete therapeutic products, offering a continuous, low‐shear, and fouling‐free alternative to conventional filtration processes.

## 1 Introduction

Lentiviral vectors (LVVs), derived from human immunodeficiency virus type 1 (HIV‐1) and engineered to be safer, self‐inactivating systems, emerged in the early 2000s as powerful gene‐delivery vehicles due to their ability to transduce non‐dividing cells and achieve stable integration of therapeutic transgenes (Mátrai et al. 2010; Naldini et al. 2016). In cell therapy, LVVs have been employed for ex vivo modification of hematopoietic stem cells (Eichler et al. 2017; Ferrari et al. 2021) and for engineering T cells, including CAR‐T therapies targeting CD19 in B‐cell malignancies (Cappell and Kochenderfer 2023; Grupp et al. 2013). More recently, efforts have focused on developing in vivo CAR‐T generation via lentiviral delivery directly to T cells, bypassing ex vivo cell manufacturing. However, despite these compelling applications, manufacturing of LVVs remains constrained by several difficulties (Perry and Rayat 2021): their high sensitivity to shear stress during bioprocessing, relatively strong negative surface charge, which promotes non‐specific binding and adsorption losses (Pamenter et al. 2025), as well as instability of the vector particles during downstream processing and storage (Higashikawa and Chang 2001; Luostarinen et al. 2024). These limitations result in significant product losses, which in turn contribute to the high cost of LVV‐based cell therapies. Vector manufacturing has become a dominant cost driver for cell and gene therapies, preventing broader access and scalability.

Overall LVV recovery through the entire downstream process has been reported to be approximately 30%–40% in conventional workflows, indicating substantial product losses across multiple purification stages (Bandeira et al. 2012). Among these, clarification with a depth filter is known to result in a 10%–25% loss of functional lentiviral vector particles, emphasizing the need for optimized recovery strategies at this stage (Bandeira et al. 2012; Valkama et al. 2020; Mayani et al. 2024; Labisch et al. 2021). Previous efforts to address this challenge are mostly based on incremental optimization of membrane processes, with mixed outcomes. Depth filtration recovery has been improved from 74% to 91% by operating at a high flow rate (Bandeira et al. 2012). It was also suggested that the increased flow rate may reduce entrapment of viral vectors within the depth filters; however, the underlying mechanism was not investigated in detail, and the need for optimization depending on the lentiviral envelope was also noted. In another study, approximately 90% functional recovery was achieved by implementing a filter‐flushing step (Valkama et al. 2020). In intensified suspension cultures (> 10^7^ cells/mL), optimizing a two‐stage depth filtration strategy increased recovery from 34% to 82% while maintaining low turbidity (≤ 20 NTU) (Mayani et al. 2024). Although flushing and two‐stage filtration are commonly used to enhance recovery during clarification, these also increase process complexity. In addition, a diatomaceous earth‐aided filtration process has been reported; however, after 0.45 µm filtration, the infectious vector yield remained at approximately 65% of that obtained with centrifugation followed by 0.45 µm filtration (Labisch et al. 2021).

Inertial microfluidics exploits hydrodynamic lift forces that arise at intermediate Reynolds numbers to focus and separate particles without the need for external forces (Martel and Toner 2014). However, the inherently low throughput of a single microchannel limits its applicability to bioprocessing, where cell suspensions at hundreds‐ to thousand‐liter scales are typically handled. To overcome this limitation, our group developed a stacked spiral inertial microfluidic system fabricated from injection‐molded plastic layers (Jeon et al. 2022, 2025). By multiplexing 100 spiral channels in parallel, we achieved liter‐per‐hour‐scale throughput for CHO cell clarification, demonstrating the scalability of inertial microfluidics. This approach highlights the potential of multiplexed spiral devices to bridge the gap between lab‐scale microfluidic throughput and industrial bioprocessing requirements.

In this study, we develop a microfluidic cell clarification (MCC) system for LVV harvest that improves cell‐clarification efficiency, reduces processing time, and enhances product recovery. In the MCC process, crude culture fluid from a recirculation flask enters the device, where cell‐focused streams are redirected back to the flask while clarified fluid is continuously harvested. By optimizing hydraulic configuration, flow conditions, and harvest strategy, the clarified fluid achieved a cell density of less than 0.2 × 10^6 ^cells/mL and a turbidity of approximately 20 NTU, down from an initial turbidity of ~100 NTU. To prevent cell breakthrough that occurs as cell concentration increases during recirculation, a buffer addition strategy was implemented, maintaining overall cell removal efficiency above 95% and LVV recovery above 95%. After 0.45 µm secondary filtration, the LVV recovery increased by 28% compared with a benchmark process (cellulose depth filter followed by a 0.45 µm PES filter). Finally, the scalability of the MCC platform was demonstrated using a 75‐stack device operating at a harvest flow rate of 1.5 L/h without performance loss. With further scale‐up, this technology is expected to offer a promising alternative for efficient cell clarification in LVV manufacturing and broader bioprocessing applications.

## 2 Materials and Methods

### 2.1 Microfluidic Device Preparation

The microfluidic device was composed of three injection‐molded components produced by a vendor (RnD Factory, South Korea): an input layer with a single port, an output layer with two outlet ports, and a channel layer. The channel layer contains a groove that defines the microfluidic channel when bonded to one side of a double‐sided silicone adhesive film (EZsolution, South Korea), while the opposite side of the film was attached to another channel or port layer. The trapezoidal cross‐section of the channel had dimensions of 200 × 140 × 1000 μm (inner depth × outer depth × width). Each device unit consisted of an input layer, 25 or more channel layers, and an output layer, which were stacked together and clamped between two aluminum plates with screws to prevent leakage. The detailed fabrication and stacking procedure was described in our previous study (Jeon et al. 2022). The difference from the previous device is that the inlet and outlet port layers are positioned on opposite sides. This configuration is designed to distribute the fluid evenly across multiple channel layers. The assembled device was sterilized by gamma irradiation (Gammacell 220 F) at a dose of 25 kGy.

### 2.2 Cell Culture and Lentivirus Production

A HEK 293 lentiviral vector producer cell line engineered to generate LV‐CMV‐GFP lentiviral vectors (a gift from the National Research Council Canada) was cultured in Hycell‐Transfx‐H medium supplemented with 4 mM l‐glutamine and 0.1% (v/v) Kolliphor P188. Lentiviral vector production was induced by the addition of 110 μg/mL cumate and 12 nM coumermycin, followed 24 h later by the addition of 7 mM sodium butyrate. The post‐induction culture was maintained for 3 days.

### 2.3 Operation of the Clarification System

**Figure 1:**
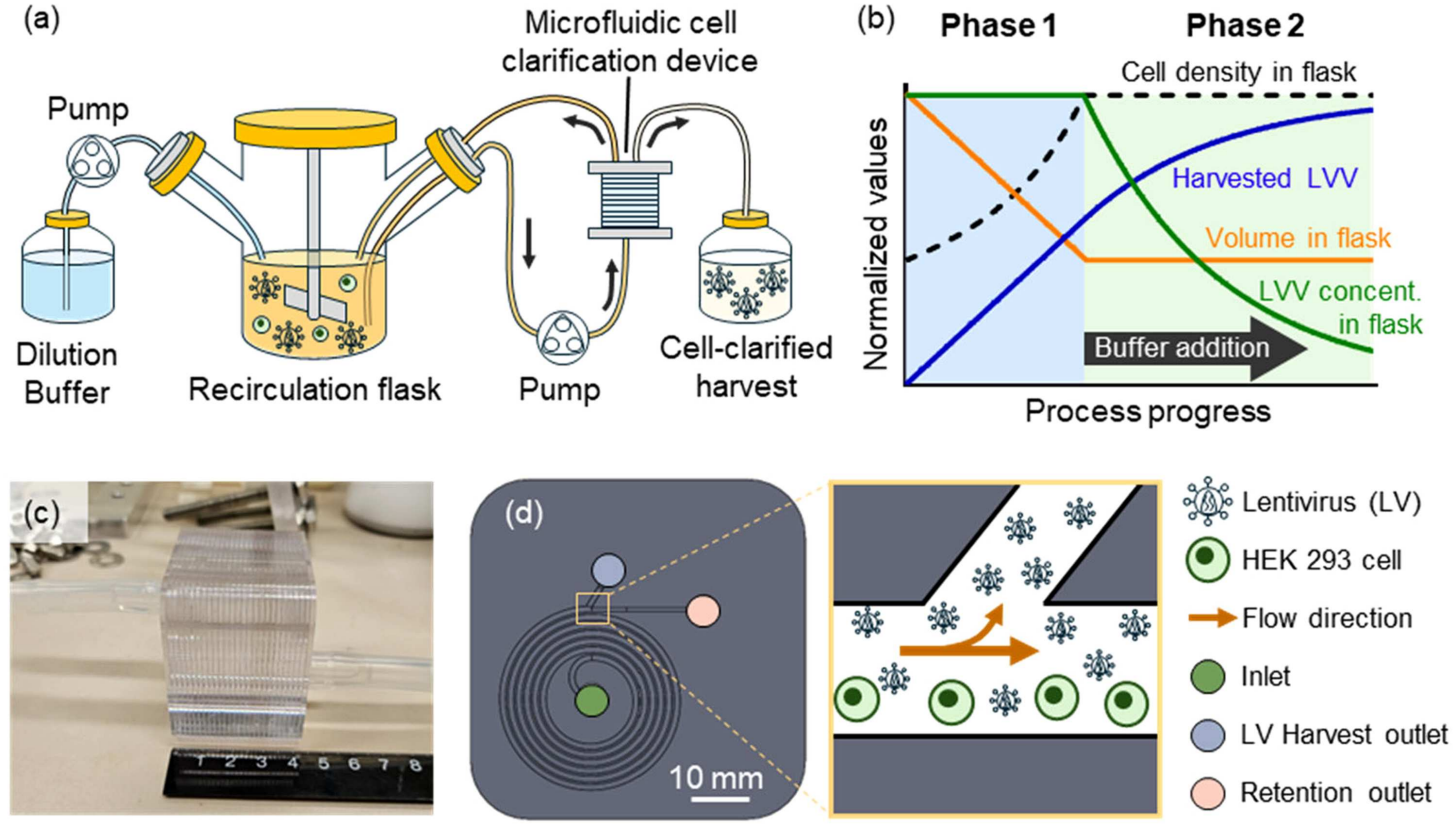
Overview and configuration of the microfluidic cell clarification (MCC) system. (a) Schematic diagram of the MCC system. A recirculation flask containing crude culture fluid is connected to the MCC device, which continuously separates cell‐clarified LVV harvest from the culture fluid. A dilution‐buffer bottle is connected to the flask to control the cell density in the recirculated culture fluid. (b) Normalized plots showing the volume in the flask, LVV concentration, and remaining LVV in the flask as the process proceeds. The light‐blue region indicates Phase 1, during which harvesting is performed without buffer addition, while the light‐green region represents Phase 2, when harvesting continues with buffer addition to maintain constant volume and cell density. The green line represents LVV concentration, the orange line represents the volume, and the black dashed line represents the cell density in the flask. Blue line indicates cumulative LVV harvest. (c) Photograph of the 25‐stack MCC device with silicone tubing connected to the inlet and outlet ports on both sides. (d) Bottom view of the microfluidic channel‐layer layout and schematic diagram illustrating LVV–cell separation at the junction region where clarification occurs.

Crude lentiviral producer cell culture fluid was prepared in a flask and introduced to a 25‐stack clarification device by a peristaltic pump (Pump head: 77202‐60, Masterflex) with a total flow rate of 125 mL/min (5 mL/min/layer). The harvest flow rate was adjusted by connecting thin tubings (ID: 0.8 mm) with different lengths to the harvest outlet, resulting in split ratios (retention outlet: harvest outlet) of 14:1 and 7.6:1. The split ratio is defined as the ratio of flow rates at the retention outlet (RO) to that at the harvest outlet (HO). Hereafter, the split ratio is expressed as RO: HO. The retentate is returned to the recirculation flask, and the harvest is collected sequentially in 50 mL tubes, which are combined after analysis of cell density and turbidity. During phase 1 operation (without buffer addition), the clarified harvest was collected until the cell density in the recirculation flask reached 1 × 10^7 ^cells/mL. At that point, phosphate‐buffered saline (PBS) was added to the flask at the harvest flow rate to maintain a constant volume in the recirculation system (phase 2). A photo of the experimental setup is provided in the Supporting Information S1: Figure 1. The operation was terminated when the theoretical harvest rate, calculated as the cumulative amount of harvested LVVs divided by the total LVVs present in the initial culture, reached 95%. Crude culture, process harvest fractions, and the pooled harvest samples were centrifuged at 300*g* for 3 min, and the resulting supernatants were cryopreserved at −80°C for further analysis.

### 2.4 Cell Density and Turbidity Measurements

Cell density was measured using a FLEX2 Cell Analyzer (Nova Biomedical), and turbidity was measured using an Orion AQUAfast AQ3010 Turbidity Meter (Thermo Scientific), according to the manufacturers' instructions.

### 2.5 Benchmark Clarification Process

As a benchmark, depth filtration followed by 0.45 µm filtration was performed. Crude LVV culture fluid was introduced to a depth filter (Millipore Sigma, Cat. # MCE5023CL3) with nominal pore sizes ranging from 0.6 to 1.3 μm and an effective area of 23 cm^2^ using a peristaltic pump. For secondary filtration, a 1.2/0.45 μm syringe filter (Millipore Sigma, Cat. #SMP4A25NB6) with an effective area of 3.5 cm^2^ was utilized. A pressure gauge (McMaster‐Carr, Cat. #1287N1) was installed between the pump and the filter to monitor the filter backpressure. The filters were primed with PBS before the culture fluid was introduced. The culture fluid was delivered at a flow rate of 300 L/m^2^·h, corresponding to volumetric flow rates of 11.5 mL/min and 1.75 mL/min through the depth filter and PES filter, respectively, until the pressure upstream of the depth filter reached 0.5 bar. The clarified fluid from the filter was collected in a bottle placed on a scale to monitor the collected volume. No additional buffer flushing step was performed after processing.

### 2.6 Functional Titer Measurement

Lentiviral vector samples collected by microfluidic clarification were stored at −80°C in either cell culture medium or a mixture of cell culture medium and PBS introduced during filter wetting and phase 2 MCC operation. Jurkat cells were cultured at 5% CO_2_ at 37°C in RPMI 1640 Medium (Thermo Fisher, USA) supplemented with 10% (v/v) Fetal Bovine Serum (FBS), 2 mM l‐Glutamine, and 1% (v/v) Penicillin‐Streptomycin. Jurkat cells were transduced in 24‐well plates in the presence of 10 µg/mL polybrene and centrifuged at 1000*g* for 1 h at 37°C. Twenty‐four hours post‐transduction, 500 μL of RPMI 1640 supplemented with 10% v/v FBS, 2 mM l‐glutamine, and 1% v/v Penicillin‐Streptomycin was added to each well. Sixty‐hours post‐transduction, the cells were resuspended in PBS containing 1% bovine serum albumin (BSA) and DAPI for live/dead cell staining. Data were acquired on the FACSymphony A3 flow cytometer (BD Biosciences, USA) using the BB515 detector for the 488‐nm laser line and FACSDiva software. Live single cells were gated using forward scatter, side scatter, and DAPI, and then GFP+ cells were quantified. Ten thousand events were recorded in the live singlets gate (BV421‐A vs FSC‐H) for each well. Further analysis was conducted using FlowJo v10 (BD Biosciences, USA). A Poisson distribution was used to describe the relationship between the percentage of GFP+ cells and the multiplicity of infection (MOI), as described in detail in our previous study (Bevacqua et al. 2026). The Poisson distribution is expressed as:

$$P\left(n\right)=\frac{\lambda^{n}e^{-\lambda }}{n!}$$

where λ is the MOI and P(n) is the probability that a cell is infected by n LVs. Uninfected Jurkat cells are described in Poisson terms as $P\left(n=0\right)=\;e^{-\mathrm{MOI}}=1-\%{\mathrm{GFP}}^{+}$, where $\%{\mathrm{GFP}}^{+}$ is the fraction of GFP+ events within the gated live singlet population, which leads to the Poisson correction $\mathrm{MOI}=\;-\ln\left(1-\%{\mathrm{GFP}}^{+}\right)$. The functional titer is calculated as:

$$\text{Functional titer}\;=\frac{C∙\mathrm{MOI}}{V}=\frac{-C∙\ln\left(1-\%{\mathrm{GFP}}^{+}\right)}{V}$$

where C is the number of cells at the time of transduction (5 × 10^5^ cells per well for this study) and V is the volume of the LVV sample added to the well. We utilized a targeted range of 1% to 25% GFP+ cells to determine functional titers, since this ensures that the Poisson correction accurately accounts for multiple LVV integration events.

### 2.7 Host Cell Protein and DNA Quantification

Host cell protein (HCP) levels were quantified using a HEK293 HCP ELISA kit (F650S, Cygnus Technologies, USA) according to the manufacturer's instructions. Absorbance was measured using a plate reader (Tecan SAFIRE, USA), and HCP concentrations were calculated from a standard curve fitted using a four‐parameter logistic (4PL) model. Host cell DNA levels were quantified using PicoGreen assay kit (P7589, Thermo Fisher Scientific, USA) according to the manufacturer's instructions. Fluorescence was measured using a plate reader with excitation/emission wavelengths of 500/525 nm. Host cell DNA concentrations were calculated from a PicoGreen standard curve within the linear detection range.

## 3 Results

### 3.1 Multiplexed Inertial Microfluidic Cell Clarification System

An inertial microfluidic cell clarification (MCC) device separates cell‐free media from cells, retaining the cells in the recirculation system, as shown in Figure 1a. Figure 1b illustrates the operational strategy for efficient LVV harvest. The operation process consists of two phases. The first phase (Phase 1) harvests cell‐free medium by recirculating the original volume without adding dilution buffer (PBS), thereby gradually increasing cell density in the recirculation flask while maintaining minimal cell breakthrough at the MCC. In the second phase (Phase 2), dilution buffer is added at the same flow rate as the harvest flow rate to maintain a constant volume and cell density in the flask, typically 10–15 × 10^6 ^cells/mL. The amount of harvested LVV increases linearly during Phase 1, whereas the rate of harvest gradually decreases during Phase 2 due to buffer addition. A 25‐stack multiplex device has a height of 43 mm, including the thickness of the plastic layers (1.5 mm each) and silicone adhesive films between adjacent layers (Figure 1c). The bottom view of a unit channel layer (57.6 mm (W) × 58.5 mm (H)) is illustrated in Figure 1d. Cells are focused along the inner spiral side, where inertial forces are balanced, while LVVs disperse over the channel due to their small size. A harvest outlet, connected to a branch channel originating on the opposite side of the focused cell stream, removes only a small fraction of the total flow compared with the retention outlet. This configuration enables selective harvesting of LVVs while minimizing cell breakthrough from the focused cell stream.

### 3.2 Clarification Efficiency According to Split Ratios

**Figure 2:**
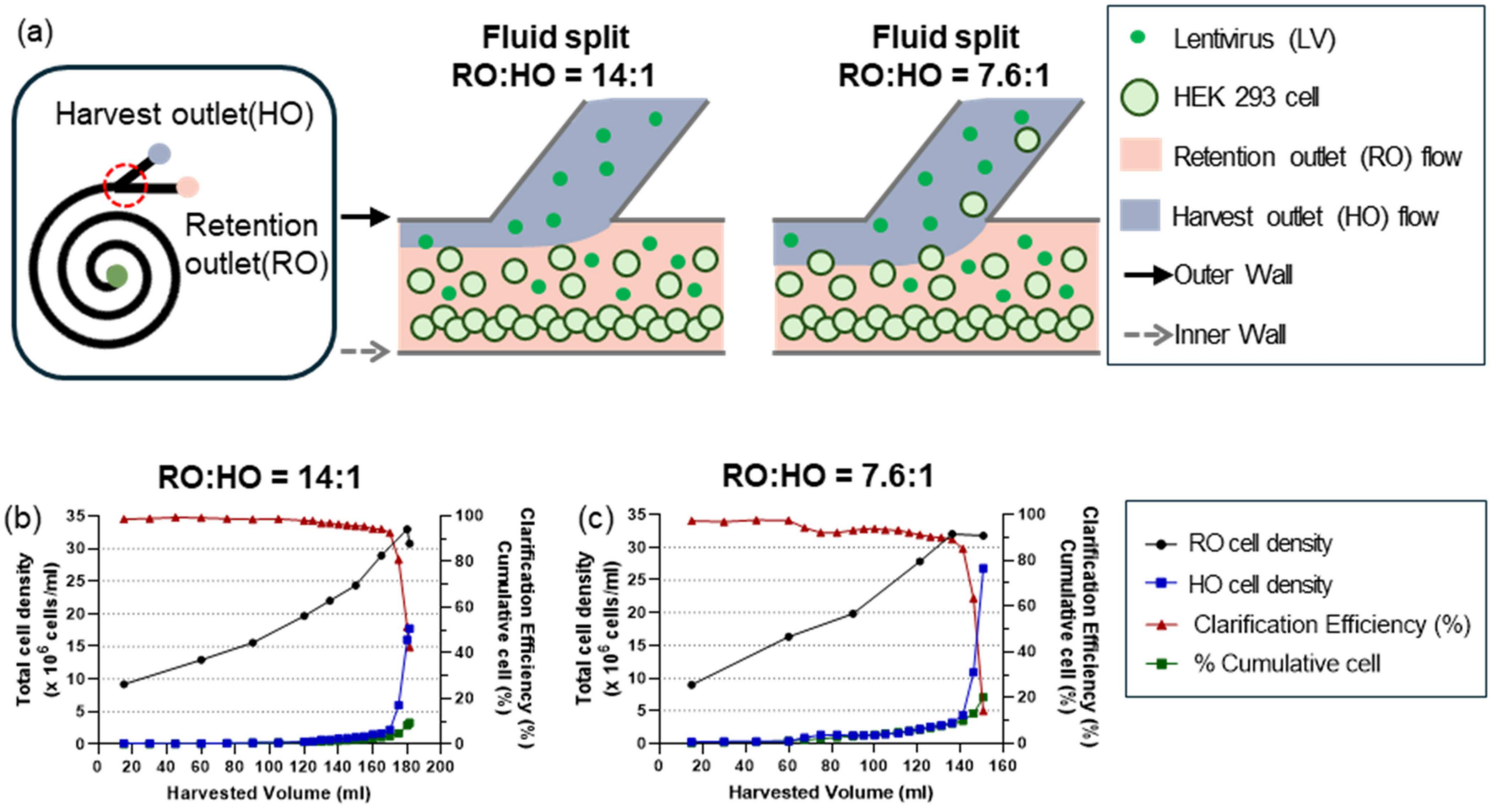
Split ratio optimization of the microfluidic cell clarification (MCC) system. (a) Schematic diagram illustrating LVV–cell separation behavior as a function of the split ratio. When the flow rate through the harvest outlet (HO) increases, stronger hydrodynamic interference occurs near the inner spiral wall where cells are focused, resulting in partial release of weakly focused cells into the HO stream. (b) Clarification efficiency profile obtained at a split ratio of 14:1. (c) Clarification efficiency profile obtained at a split ratio of 7.6:1. In (b, c), black circles indicate the cell density at the retention outlet (RO) during processing, blue squares represent the cell density at the harvest outlet (HO), red triangles show the instantaneous clarification efficiency, and green squares denote the cumulative percentage of cells recovered through the HO relative to the initial cell amount in the flask.

As the clarification run progresses, cell density in the recirculation flask gradually increases, resulting in a thicker cell‐focused stream and a higher risk of cell breakthrough at the harvest outlet. To determine the transition point at which buffer addition begins to prevent cell breakthrough through the harvest outlet, we tested clarification efficiency at two split ratios. Figure 2a illustrates how split ratios affect the clarification efficiency. We prepared a crude culture fluid, operated the clarification device, and sequentially collected samples from the harvest outlet and the flask. As clarified samples are collected, the cell density in the flask increases, and the clarification efficiency is calculated as [1‐CD_H_/CD_F_] × 100(%), where CD_H_ is the cell density at the harvest outlet, and CD_F_ is the cell density in the flask. When HO takes a small fraction of the flow (14:1 split ratio), the cells barely enter the harvest stream. In contrast, cells escape through HO at a higher flow rate toward HO (7.6:1 split ratio). Figure 2b,c shows the clarification efficiency at split ratios of 14:1 and 7.6:1, respectively. The input flow rate was 125 mL/min (5 mL/min/layer) in both conditions. For the 14:1 split ratio, the initial cell density of crude cell culture was 9.2 × 10^6^ cells/mL, and the volume was 250 mL. Until the CD_F_ reached 2 × 10^7^ cells/mL, CD_H_ was maintained below 0.5 × 10^6^ cells/mL. The clarification efficiency remained above 95% until the flask cell density reached 2.5 × 10^7^ cells/mL. In the meantime, the higher flow rate toward HO (i.e., lower split ratios) resulted in significant cell breakthrough (< 95% clarification efficiency) at a lower cell density (> 1.6 × 10^7^ cells/mL). Generally, the clarification efficiency remained above 90% until the CD_F_ reached 2.9 × 10^7^ cells/mL, setting an upper bound for the clarification operating conditions. In the following experiments, we used a 14:1 split ratio (harvest flow rate of 8.3 mL/min with a 25‐stack device) to minimize cell breakthrough into the harvest.

### 3.3 Cell Clarification Efficiency and LVV Harvest Efficiency

**Figure 3:**
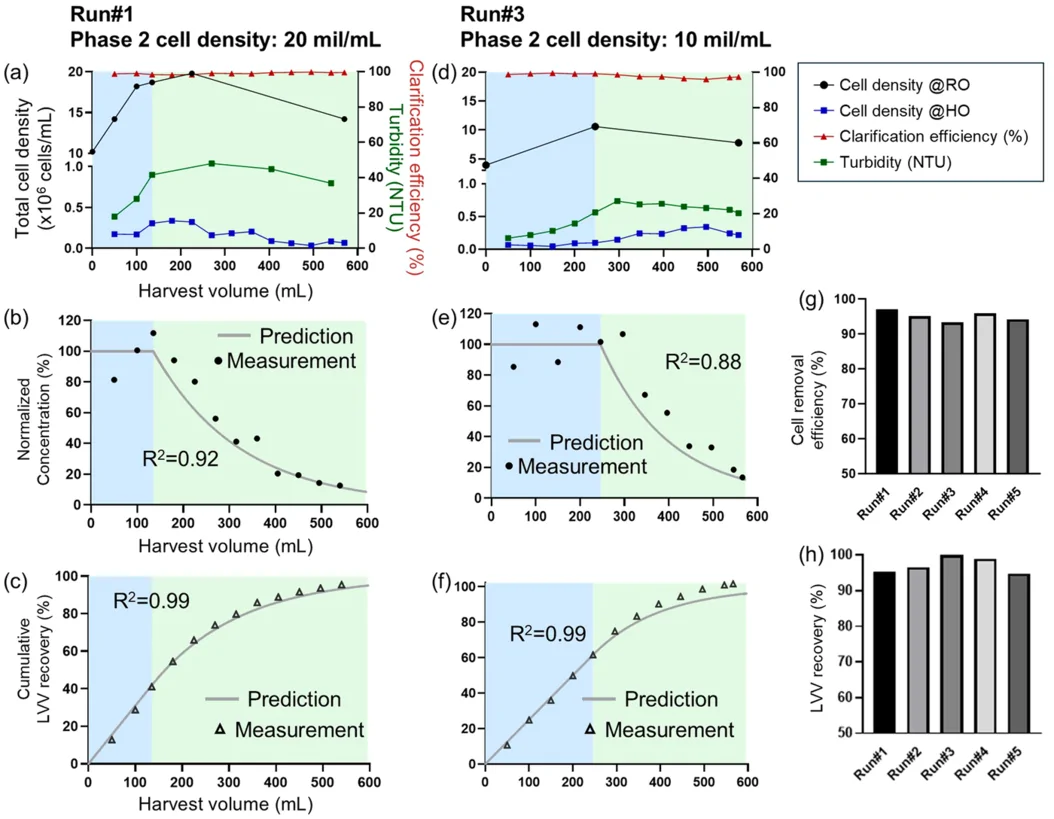
Cell removal efficiencies and LVV harvest yields across multiple crude material conditions and Phase 2 cell densities. (a–c) Cell clarification and LVV harvest performance at a Phase 2 cell density of 20 × 10^6^ cells/mL. (a) Temporal profiles of cell density at the retention outlet (RO, black circles), cell density at the harvest outlet (HO, blue squares), clarification efficiency (red triangles), and turbidity (green squares) during processing. (b) Comparison between the normalized theoretical LVV concentration (gray line, assuming no LVV loss) and the measured functional titer (black circles). (c) Cumulative amount of LVV harvested during the process, expressed as a percentage of the initial LVV in the crude material. (d–f) Results obtained at a Phase 2 cell density of 10 × 10^6^ cells/mL, presented in the same format as panels (a–c). (g) Cell removal efficiencies from five independent MCC processes conducted under different initial and Phase 2 cell density conditions. (h) Corresponding LVV harvest yields from the same five MCC processes.

To minimize cell breakthrough and maximize LVV recovery efficiency, we developed a strategy in which harvesting was continued until just before substantial cell breakthrough began, at which point additional buffer was added to maintain a constant volume and cell density within the recirculation flask. To determine the optimal cell density for initiating buffer addition, we tested several conditions as shown in Figure 3. The period before buffer addition was defined as Phase 1, and the period during which buffer was added while harvesting was defined as Phase 2.

Figure 3a–c show representative results when the initial CD_F_ was 10.2 × 10^6^ cells/mL and the target Phase 2 CD_F_ was 20 × 10^6^ cells/mL (Run#1). Immediately after buffer addition began, a slight increase in cell density in the flask was observed, followed by a gradual decrease over time. The CD_H_ remained below 0.5 × 10^6^ cells/mL, while turbidity was maintained at approximately 40 NTU throughout Phase 2. The cell clarification efficiency remained above 95%. The harvest was collected sequentially in approximately 50 mL fractions, and the functional titers of each fraction were quantified using a Jurkat cell transduction assay, as shown in Figure 3b. The first three fractions were collected without buffer addition (Phase 1), and subsequent fractions, collected during buffer addition (Phase 2), showed a gradual decrease in titer, consistent with dilution. Assuming no LVV loss during processing, we modeled the LVV concentration decay due to buffer addition as *C*(*V*_h_)= $C_{0}\times e^{-\frac{V_{h}-V_{1}}{V_{2}}}$, where *C*_0_ is the initial LVV concentration, *V*_h_ is the cumulative harvest volume, *V*_1_ is the harvest volume during phase 1, and *V*_2_ is the flask volume during phase 2. The measured titers showed excellent agreement with the theoretical curve (*R* ^2^ = 0.92). Figure 3c shows the cumulative yield, which closely follows the theoretical curve for complete recovery without loss. In Run #1, the process was terminated after recovering 92.8% of the theoretical LVV amount, and the experimentally measured recovery was 95.5%, demonstrating high consistency between the theoretical and measured yields.

Similarly, Figure 3d–f present the results of another run (Run #3) in which the Phase 2 CD_F_ was 10 × 10^6^ cells/mL. The average CD_H_ was 0.18 × 10^6^ cells/mL, and the average turbidity was 19.0 NTU (Figure 3d). As observed in Run #1, both the concentration decay and cumulative yield closely matched the theoretical predictions (Figure 3e,f), confirming that our MCC system operates with negligible LVV loss. In a benchmark test using a depth filter (nominal pore size: 0.6–1.3 μm), the filtrate harvested when the pressure reached 0.5 bar exhibited a turbidity of 15–20 NTU, which was comparable to that observed at a Phase 2 CD_F_ of 10 × 10^6^ cells/mL. Therefore, in the subsequent experiments (Runs #4 and #5), Phase 2 was set to begin at CD_F_ = 10 × 10^6 ^cells/mL for direct comparison with the depth filter benchmark process. Across five independent runs with various initial and Phase 2 cell density conditions, the cell removal efficiency, [1 − (Total number of cells from HO/Total number of cells in the initial flask)] × 100(%), and LVV recovery remained highly consistent. On average, processes designed to recover approximately 95% of the total LVV achieved 97.5% apparent LVV recovery and 95.1% cell removal efficiency, as summarized in Figure 3g,h. Detailed process conditions for each run are listed in Table 1.

**Table 1:** Details of experimental conditions and performance metrics for five MCC operations.

ID	Run#1	Run#2	Run#3	Run#4	Run#5
Initial cell density (×10^6^ cells/mL)	10.2	5.4	3.9	4.5	4.3
Crude volume (mL)	323	1200	400	600	600
Phase 2 cell density (×10^6^ cells/mL)	20	15	10	10	10
LVV recovery (%)	95.5	95.5	95.2^a^	99.0	94.8
Measur LVV recovery (%)				99.0	94.8

^a^ Due to variability inherent to cell‐based transduction assays, Run #3 resulted in an apparent yield exceeding 100%.

### 3.4 Benchmarking With a Conventional Depth Filter

**Figure 4:**
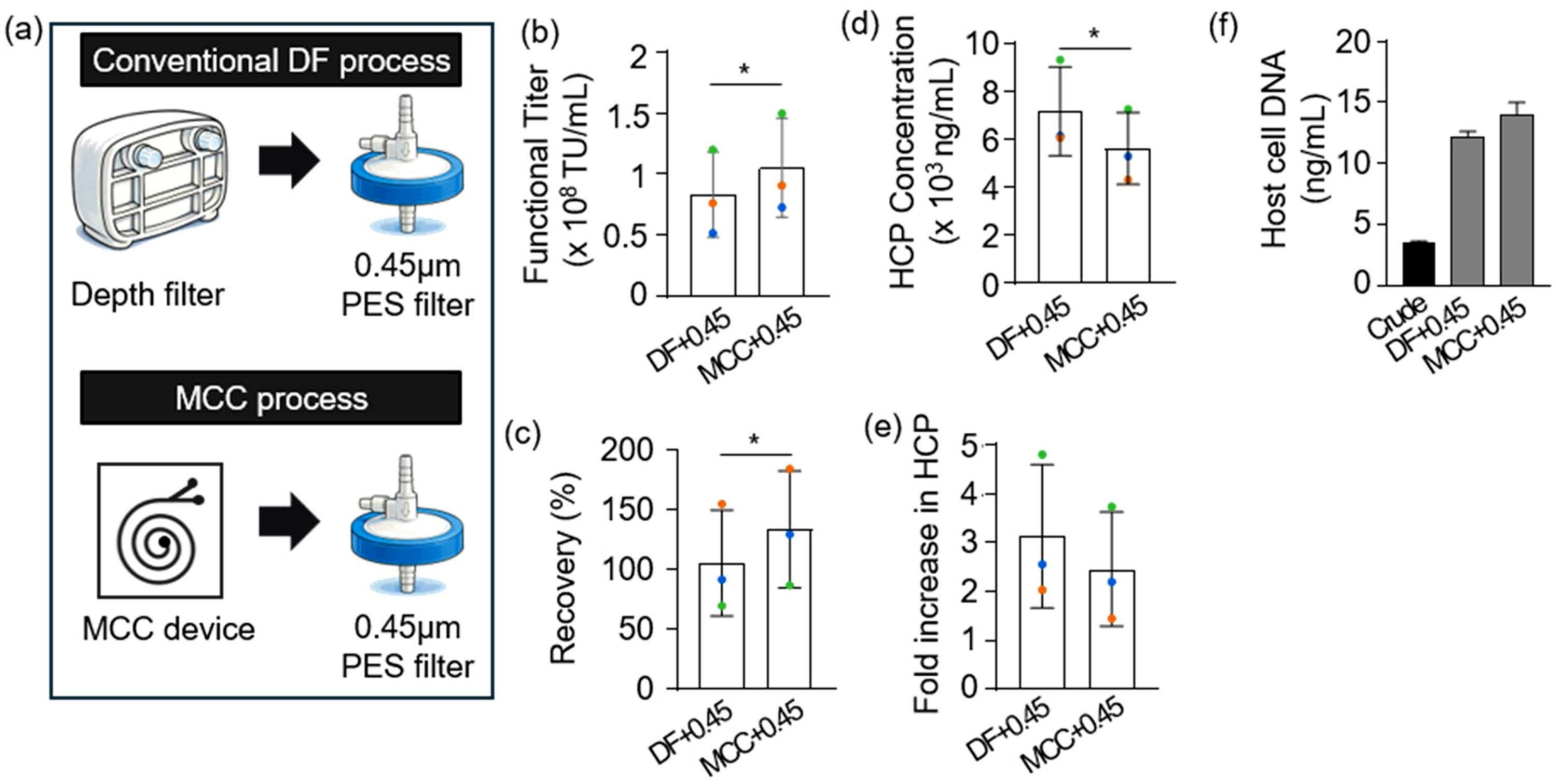
Comparison of the MCC process with the conventional depth filtration (DF) process. (a) Schematic diagrams of the DF process and the MCC process. Both processes included a secondary 0.45 µm PES filtration step following the primary clarification step. (b) Functional titers after the DF and MCC processes, normalized to the initial sample volume (harvested TU per initial volume). Each dot represents the mean value from triplicate transduction wells within a single batch, while the bars represent the average of three independent batch operations. Error bars indicate standard deviation. Dots with the same color represent results from the same batch. (c) Percent recovery of LVV from the DF and MCC processes. (d) HCP concentrations after the DF and MCC processes, normalized to the initial sample volume. Each dot represents the mean value from triplicate ELISA wells within a single batch, the bars represent the average of three independent batch operations, and error bars indicate standard deviation. (e) Fold increase in HCP concentration relative to the crude material after the DF and MCC processes. (f) Host cell DNA concentrations after DF and MCC processes. Bars represent the mean value from triplicate Picogreen assay wells. Normalized to the initial sample volume. Statistical significance was determined using a paired *t*‐test with batch triplicates (*p < 0.05).

Because LVVs are highly sensitive to shear stress and possess a relatively high negative surface charge, many manufacturing processes employ depth filtration for primary clarification, followed by a 0.45 μm polyethersulfone (PES) membrane filtration. A product loss of approximately 20% has been reported at each filtration step (Bandeira et al. 2012; Valkama et al. 2020; Mayani et al. 2024; Labisch et al. 2021; Vogel et al. 2024). To benchmark our system, we compared the performance of the MCC process followed by 1.2/0.45 μm PES filtration (MCC process) with a conventional clarification process consisting of depth filtration (nominal pore size 0.6–1.3 μm) followed by 1.2/0.45 μm PES filtration (DF process) as shown in Figure 4a. LVV‐producing HEK cell cultures were pooled and evenly split into two identical portions for parallel DF and MCC processing. For example, 8 flasks containing 100 mL of culture were combined and divided into two 400 mL flasks. Three independent culture batches were evaluated for the parallel comparison of the DF and MCC processes.

Depth filtration was operated until a critical pressure of 0.5 bar was reached, resulting in partial consumption of the crude volume, whereas the spiral‐based MCC process fully processed the entire allocated volume. LVV titers were measured after completion of each process, including secondary filtration. To account for dilution effects arising from buffer addition during the MCC process and filter priming, titers were normalized to the initial sample volume and reported as harvested transduction unit (TU) per initial volume. Using this experimental design, three independent experiments were performed to compare the MCC process with the DF process. Across the three runs, the MCC process exhibited significantly higher final titers than the DF process (Figure 4b), resulting in apparent recoveries exceeding 100% on average (Figure 4c). In some runs, LVV recovery substantially exceeded 100%, likely reflecting variability and uncertainty in crude suspension titer measurements. Similarly, when HCP concentrations were normalized to the initial culture volume, the MCC process showed a smaller increase in HCP levels than the DF process (Figure 4d). Across three independent experiments, HCP levels increased by 3.1‐fold and 2.5‐fold after the DF and MCC processes, respectively (Figure 4e). In one representative batch experiment, host cell DNA levels were monitored throughout the clarification process. Consistent with the known adsorption properties of depth filters toward host cell DNA, the DF process resulted in a lower final host cell DNA concentration than the MCC process (Figure 4f). Nevertheless, the host cell DNA concentration after the MCC process remained within a range that can be readily addressed during downstream processing.

### 3.5 Scalability Test With a 75‐Stack Microfluidic Cell Clarification Device

**Figure 5:**
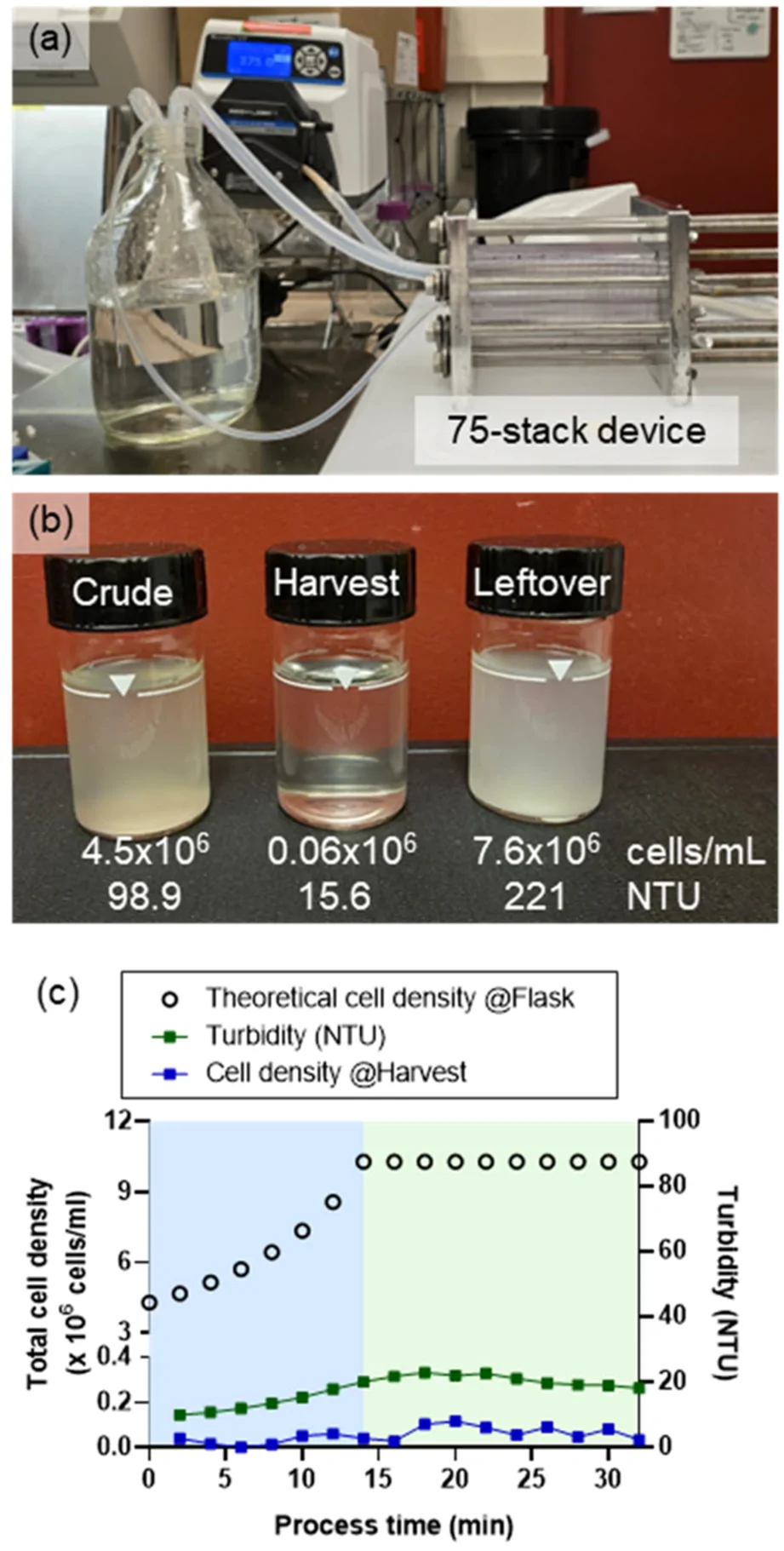
Operation of the 75‐stack microfluidic cell clarification (MCC) device. (a) Experimental setup for the 75‐stack device operation. The initial cell density in the flask was 4.5 × 10^6^ cells/mL, and the total crude‐material volume was 600 mL. The photograph on the right shows the 75‐stack device with the buffer bottle used during the flow test, which was replaced by the flask containing crude cell material before the experiment began. (b) Comparison of turbidity among crude, harvested, and recirculated fluids. From left to right, the vials contain crude culture fluid (98.9 NTU), pooled harvest fractions (15.6 NTU), and the residual fluid (221 NTU) remaining in the recirculation flask after the run. (c) Temporal profiles of the theoretical cell density at the recirculation flask (open circles), the measured cell density at the harvest outlet (HO, blue squares), and turbidity (green squares) during processing.

The results shown in Figure 4 were obtained using a 25‐stack MCC device with optimized process conditions: an input flow rate of 125 mL/min and a harvest flow rate of 8.3 mL/min. To further evaluate the system's scalability, we processed 600 mL of crude material at an initial cell density of 4.3 × 10^6^ cells/mL using a 75‐stack MCC device, as shown in Figure 5a. The harvest flow rate increased threefold to 24.9 mL/min relative to the 25‐stack setup, reducing the harvest time to 32 min to harvest 850 mL, including buffer addition. Despite the higher flow rate, the CD_H_ remained below 0.2 × 10^6^ cells/mL, and turbidity was maintained at approximately 20 NTU, indicating clarification performance comparable to that of the 25‐stack device (Figures 5b,c). These results confirm the scalability of the MCC system without loss of clarification efficiency and indicate its potential for rapid recovery of labile lentiviral vectors, thereby minimizing degradation during harvest.

## 4 Discussion

In this study, we realized a medium‐throughput inertial microfluidic cell clarification (MCC) device that achieved over 95% LVV recovery. The MCC device recovers the product by continuously withdrawing cell‐free fluid from the opposite side of the inertially focused cell stream while recirculating the concentrated cells. As a result, the cell concentration in the recirculation loop gradually increases, eventually causing cell breakthrough beyond a threshold. This can be prevented by maintaining a lower cell density through buffer addition or by reducing the harvest flow rate. In this study, buffer was added during operation to maintain the recirculated cell density below the breakthrough threshold until 95% LVV recovery was achieved. Although extended operation with additional buffer supply could further improve cumulative product recovery, buffer addition may dilute the LVV‐containing harvest and increase the burden on downstream tangential flow filtration (TFF)‐based concentration steps. Conversely, for processes where throughput is prioritized, early termination can reduce buffer consumption and processing time. Additionally, buffer addition can be reduced by adjusting the split ratio and reducing the harvest flow rate at higher cell densities. In this study, harvesting was performed at a constant flow rate, but a dynamic harvesting strategy could be implemented in future work. For example, the system could harvest more rapidly during the initial low‐cell‐density phase and then gradually reduce the harvest rate as cell density increases. Such an approach could maintain overall throughput while significantly reducing the amount of buffer required. Overall, these results suggest that the MCC process could serve as a drop‐in replacement for conventional depth filtration in lentivirus production, if properly scaled to manufacturing‐scale volumes.

**Figure 6:**
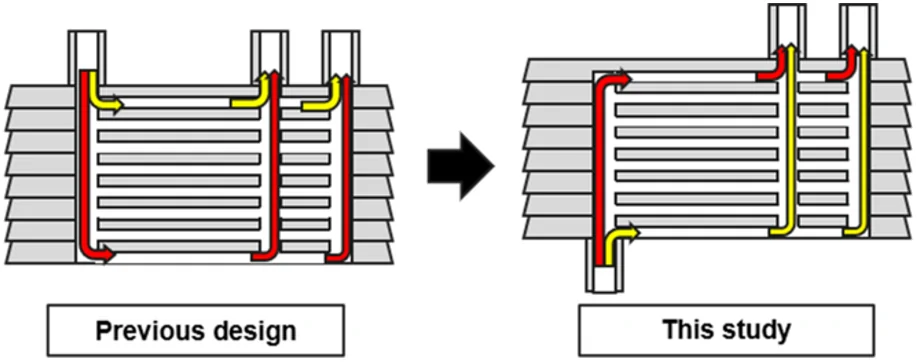
Schematic diagram of flow distribution depending on the port configuration. The old device had both inlet and outlet ports on the same side, whereas the device used in this study has the inlet and outlet ports on opposite sides. This modification made the flow path length from the inlet to the outlet equivalent across all stacked layers, thereby enabling uniform flow distribution to each layer. Yellow arrows indicate the flow path through the first layer after entering from the inlet, while red arrows represent the flow path through the final layer.

While inertial microfluidic cell retention and clarification have been reported (Jeon et al. 2022; Bevacqua, Liu, et al. 2026; Kwon et al. 2017), this work advances MCC technological readiness and reliability by implementing several critical engineering modifications, as shown in Supporting Information S1: Figure S1. Firstly, the inlet–outlet design of the stacked system is redesigned and optimized to prevent multiple stacks from inducing flow‐path differences among layers. The concept of uniform flow distribution is shown in Figure 6. Secondly, adopting a single spiral channel per layer prevented the uneven flow distribution observed in the previous 2 × 2 channel configuration (Bevacqua, Park, et al. 2026), which was attributed to clogging in one of the branches. Thirdly, pressure monitoring and fluidic stability control ensure reliable and predictable clarification performance (Jiao et al. 2019). Finally, the adoption of rigid, non‐deformable, and manufactured plastic spiral channels significantly reduces the issues associated with channel deformation (Jeon et al. 2022), clogging, and other challenges observed in previous implementations with polydimethylsiloxane (PDMS) devices. These updates significantly enhanced the purity of the harvest compared with previous implementations and enabled reliable, repeatable, and stable operation of high‐throughput spiral channel stacks for MCC, achieving the highest cell clarification efficiency ever.

In a previous study using depth filters, distributing the filtration load across sequential filtration stages has been shown to mitigate pressure buildup and significantly improve functional lentiviral recovery compared with single‐stage filtration, indicating that mechanical stress–induced damage contributes to vector loss during filtration (Mayani et al. 2024). Increased transgene length has also been associated with reduced lentiviral packaging efficiency and infectious titers, which is commonly attributed to compromised genome integrity and reduced vector robustness (Sweeney and Vink 2021). Vectors encoding larger expression cassettes are therefore expected to be structurally more fragile and less tolerant to external stresses. Filtration‐based downstream processing exposes lentiviral particles to mechanical stress and repeated contact with membrane surfaces, potentially leading to a loss of infectivity. Taken together, these observations suggest that vectors carrying longer transgenes may be more susceptible to functional inactivation during filtration due to reduced structural stability and increased sensitivity to shear and interfacial stresses. Although the DF process used in this study was not fully optimized, we observed in internal experiments that operating the filtration process beyond the selected critical pressure of 0.5 bar resulted in further reductions in functional LVV titers consistent with previous observations (Mayani et al. 2024). We recognize that additional optimization of membrane‐based clarification processes, including filter selection, membrane area, staging strategy, and operating conditions, could potentially improve recovery performance. However, such optimizations may also involve tradeoffs in process complexity, dead volume, or material cost. By contrast, the MCC platform employs channel dimensions that are at least 1000× larger than the characteristic size of lentiviral particles. As a result, LVV clarification is expected to be less sensitive to transgene length. Moreover, the process operates under stable pressure conditions without significant pressure buildup, which may enable more consistent and predictable recovery of lentiviral vectors. Although further studies will be required to validate this hypothesis, these features suggest that the MCC platform could provide a relative advantage for vectors carrying larger genetic payloads compared with conventional membrane‐based filtration.

In addition to conventional depth filters, several alternatives for harvesting LVVs from host cells include tangential flow depth filtration (TFDF) (Tona et al. 2023), counterflow centrifugation (Chaluvappa et al. 2024), and an acoustic filter (Klimpel et al. 2023). The TFDF system is designed as a cell retention device that enables continuous harvesting of LVVs during perfusion culture. This approach demonstrates the potential for process intensification in LVV manufacturing. However, the recovery rate tends to decrease under high‐cell‐density conditions, primarily due to filter fouling. Counterflow centrifugation offers a low‐shear, cell‐density‐independent, and scalable alternative for LVV harvest. Nevertheless, its complex mechanical operation increases equipment and maintenance costs, and reliance on expensive single‐use consumables limits the system's accessibility. Acoustic filtration is another fouling‐free, low‐shear technique that supports continuous LVV production. Yet it also requires a sophisticated, costly acoustic chamber and, like the MCC device, exhibits limitations in handling very high cell densities. In summary, alternative technologies beyond conventional depth filtration generally require expensive equipment and demand substantial capital investment from manufacturers. In addition, the regulatory preference for single‐use materials in viral vector production further increases operational costs. As a result, despite potential yield improvements, these economic and regulatory factors create significant barriers to the industrial adoption of such technologies.

Compared with depth filters, the MCC device has a much smaller surface area and is free of fouling, preventing backpressure buildup during operation. In addition, because the channel dimensions are significantly larger than the pore sizes used in conventional filters, shear‐induced damage to the lentiviral vectors is minimized. Although adsorption of proteins or LVV particles to the MCC device was not directly quantified in this study, no detectable deterioration in separation performance was observed during the approximately 1 h processing period. Furthermore, no significant product loss attributable to the device was observed during processing. In fact, the ~5% loss of LVV in our process is most likely due to LVV remaining in the original volume, which can be recovered by adding more buffer volume and processing it with the MCC. Consistent with this interpretation, our theoretical prediction of real‐time product recovery showed excellent agreement with experimental measurements, and the remaining volume and the expected leftover LVVs closely matched the measured decrease in total LVV titers during harvest. Although apparent LVV recovery occasionally exceeded 100%, this is likely attributable to variability in crude suspension titer measurements. One possible explanation is that crude LVV samples containing high levels of proteins and cell debris may have experienced accelerated LVV degradation during freeze–thaw cycles compared with clarified samples, resulting in underestimation of the initial crude sample titers.

The MCC device offers two major advantages: minimal product loss and compatibility with continuous operation. Because cells are continuously recirculated, fouling does not occur, allowing the system to operate at a stable pressure of approximately 100 kPa. As long as sterility is maintained, culture fluid is continuously supplied, and cell density is properly controlled, the device can be operated over an extended period without manual intervention. In fact, we have previously utilized this device as a cell retention module for the perfusion culture of CHO cells for over 15 days (Kwon et al. 2017). A limitation of the system is that complete cell removal cannot be achieved, resulting in a slight increase in the burden on secondary filtration. However, this issue can be mitigated by sacrificing throughput, making it adjustable through process optimization. The current 25‐stack device achieves a throughput of 0.5 L/h, which is still insufficient for large‐scale manufacturing. To improve throughput, the device was scaled up to a 75‐stack configuration, where it maintained its performance. Stacking up to 100 layers is expected to be feasible, as we demonstrated with the microplastic removal device (Jeon et al. 2025), which achieved a throughput of 7.5 L/h. Beyond that, parallel integration of multiple stacked cassettes is expected to enable processing capacities of several hundred liters per hour.

While this study demonstrated MCC clarification using LVV harvesting from HEK293 cell culture solutions, we believe MCC could be used for many other use cases in which biologics products are released into cell supernatant, such as monoclonal antibodies from CHO cells, certain AAV processing from HEK293 and other host cells, and many others.

## 5 Conclusion

We demonstrated that the inertial microfluidic cell clarification (MCC) device achieved over 95% cell removal efficiency and lentiviral vector (LVV) recovery. By controlling the harvest flow rate and adjusting the recirculated cell density through buffer addition, we established the relationship between clarification efficiency and throughput. Under the operating conditions tested in this study, the MCC process closely followed the theoretical recovery model, assuming negligible product loss within the device. Although the measured recovery occasionally exceeded 100%, likely due to inaccuracies in crude suspension titer measurements, the MCC process still achieved 28% higher recovery than the benchmark depth filtration process. Scalability testing further demonstrated stable clarification performance at flow rates of 1.5 L/h, supporting the feasibility of further scaling toward manufacturing applications.

Beyond LVV clarification, the MCC platform may have broader utility in bioprocessing applications involving products secreted into culture supernatants, including monoclonal antibodies, extracellular vesicles, and other viral vector modalities. In addition, because the MCC device continuously recirculates viable cells without membrane fouling, the platform could potentially serve not only as a clarification system but also as a cell retention device for perfusion culture applications.

At the same time, several important limitations and remaining challenges should be acknowledged. Although scale‐up through increased parallelization improved throughput, achieving manufacturing‐scale processing rates may require substantially higher total flow rates. Under such conditions, increased shear exposure from pumps and fluid handling systems could potentially affect both cell viability and LVV stability, particularly for structurally fragile vectors carrying larger genetic payloads. Because LVVs rapidly lose infectivity during prolonged processing, clarification is generally required within 1–2 h after harvest, making high‐throughput operation particularly important. Future work will therefore focus on further scale‐up using low‐shear pumping strategies and optimized fluidic architectures to minimize process‐induced damage during high‐throughput operation.

Additional studies will also be needed to better understand the long‐term operational stability of the MCC platform. Although we previously demonstrated stable operation of the device for more than 15 days during CHO perfusion culture, the effects of extended use, reuse, protein adsorption, and gradual performance deterioration during LVV clarification remain incompletely understood and should be systematically evaluated in future studies.

Finally, future process development efforts will focus on automated operation and dynamic process control. Real‐time adjustment of harvest flow rates and split ratios based on recirculated cell density may enable reduced buffer consumption, minimized product dilution, and improved process consistency. We believe these advances could further improve the practicality of MCC as a scalable and flexible clarification platform for viral vector manufacturing and other bioprocessing applications.

## Author Contributions

Dohyun Park, Alexander Bevacqua, Mingyang Cui, and Jongyoon Han conceptualized the study. Dohyun Park designed and fabricated the MCC platform, carried out the clarification experiment, as well as host cell protein analysis. Dohyun Park wrote the draft manuscript. Alexander Bevacqua measured the functional titers using a transduction assay. Marina Nichols and Mingyang Cui helped perform some of the experiments. Jongyoon Han and Jianzhu Chen supervised the study and revised the manuscript.

## Conflicts of Interest

The authors declare no conflicts of interest. Some authors (J.H., D.P., A.B., and M.C.) filed a technology disclosure and patents on a related technology, which are owned and managed by MIT.

## Supporting information

Supporting File

## Data Availability

The data that support the findings of this study are available from the corresponding author upon reasonable request.
