# Supplementary material for: Fouling‐Free Inertial Microfluidic Cell Clarification for Lentivirus Manufacturing
Source: Biotechnol Bioeng. 2026 Jun 7;123(9):2342–52. doi: 10.1002/bit.70262 (PMC13521457; doi:10.1002/bit.70262)
Supplement: Supplementary file 1 [file BIT-123-2342-s001.docx]

**Fouling-Free Inertial Microfluidic Cell Clarification for Lentivirus Manufacturing**

Dohyun Park^1^, Alexander Bevacqua^1,2,3^, Mingyang Cui^1^, Marina Nichols^4^, Jianzhu Chen^3^, and Jongyoon Han^1,2,5*^

^1^ Research Laboratory of Electronics, Massachusetts Institute of Technology, Cambridge, MA 02139, USA

^2^ Department of Biological Engineering, Massachusetts Institute of Technology, Cambridge, MA 02139, USA

^3^ Koch Institute for Integrative Cancer Research, Massachusetts Institute of Technology, Cambridge, MA 02139, USA

^4^ Department of Physics, Boston College, Chestnut Hill, MA 02467

^5^ Department of Electrical Engineering and Computer Science, Massachusetts Institute of Technology, Cambridge, MA 02139, USA

* Corresponding Author: Jongyoon Han (jyhan@mit.edu)

**Table of Contents**

**Figure S1.** Experimental setup of the MCC system.

**Figure S1.** Experimental setup of the MCC system. The recirculation flask containing the crude culture fluid was placed on a magnetic stirrer to prevent cell sedimentation and on a scale to monitor the volume. A peristaltic pump delivered the culture fluid to a 25-stack MCC device through a fluidic damper and a pressure gauge. The cell-concentrated stream returned to the flask, while the clarified medium was collected in harvest tubes.
